# Digital media use and cognitive networks in medical students: linking screen time with intelligence and academic performance

**DOI:** 10.3389/fmed.2026.1736060

**Published:** 2026-02-25

**Authors:** Alejandro Hernández-Chávez, Julian B. Uriarte-Ortiz, Antonio Barajas-Martínez, Raúl Sampieri-Cabrera

**Affiliations:** 1 Laboratory of Prospective, Department of Physiology, Faculty of Medicine, National Autonomous University of Mexico, México City, Mexico; 2 Tecnológico de Monterrey, School of Medicine and Health Sciences, México City, Mexico

**Keywords:** academic performance, attention, crystallized intelligence, digital media use, fluid intelligence, intelligence, medical education, network analysis

## Abstract

**Introduction:**

Digital media use has expanded rapidly among medical students, raising concerns about its impact on cognitive function and academic achievement. However, specific links between screen time and distinct facets of cognition (such as fluid vs. crystallized intelligence), as well as their combined impact on academic performance, remain underexplored in medical education.

**Purpose:**

This study explored how digital screen time and related behaviors are associated with different dimensions of intelligence (fluid and crystallized), attention, and academic performance in second-year medical students, using a network analysis approach.

**Methods:**

A cross-sectional sample of 305 second year medical students from the Faculty of Medicine, UNAM, recruited during the 2023 academic year, completed standardized assessments: the Shipley-2 test for crystallized (Gc) and fluid intelligence (Gf), the Youth Screen Time Survey, and official academic records (standardized Physiology course exam scores). Network analysis was implemented through the *igraph* package in R to identify structural relationships among 34 variables representing cognitive, behavioral, and academic domains.

**Results:**

The network analysis identified six clusters of interrelated variables, revealing distinct groupings linking digital behavior, cognitive abilities, and academic outcomes. Key variables with the highest connectivity (centrality) were the number of failed courses, total screen time, and age, indicating these factors are most influential in the network. Notably, text messaging and short-form video use (e.g., TikTok) emerged as *bridge* nodes connecting digital media use to academic performance. Higher screen time was associated with lower academic performance (*r* ≈ −0.24) and reduced fluid intelligence, while crystallized intelligence appeared relatively unaffected.

**Discussion:**

Excessive digital exposure was associated with weaker cognitive efficiency and academic performance in this cross-sectional sample. These findings underscore the need for balanced digital habits to support attention, learning, and problem-solving capacity in medical students.

## Introduction

Electronic media are extensively used in higher education, particularly among medical students (1). However, there is a growing concern that excessive screen time may impair cognitive function, intelligence, and attention, which are critical for academic performance (2, 3). Intelligence has been conceptualized through various models and approaches, one of which is described as crystallized intelligence (Gc), which reflects accumulated knowledge, and fluid intelligence (Gf), which is associated with problem-solving and adaptability (4). These cognitive abilities are closely linked to the information processing theory, particularly the Atkinson-Shiffrin multi-store model of memory (5). In this model, sensory memory acts as a temporary buffer for incoming information, filtering relevant stimuli for further processing, short-term memory processes, and manipulation of this information, thus facilitating problem solving and decision making, which are crucial for Gf (6). Frequent digital distractions could disrupt these memory processes—if constant stimuli flood sensory memory and divide attention, less information is encoded into short-term memory or consolidated into long-term memory (7). Over time, this may impair problem-solving efficiency (affecting Gf) and limit the accumulation of knowledge (affecting Gc), ultimately hindering learning outcomes (8). Finally, long-term memory serves as a repository for accumulated knowledge, directly influencing Gc (5).

Individuals spend an average of 6 h and 40 min per day on screen time. In particular, those aged 16–24 years, can spend up to 7.5 h daily, predominantly on smartphones, which are owned by 80% of the population worldwide (9). In the United States, the daily screen time has remained consistent at approximately 7 h since 2021, and nearly 80% of people aged 15–24 years are Internet users (10), and the overall screen time has increased significantly since the onset of the COVID-19 pandemic. In Mexico, data indicate that individuals aged 18–24 have the highest usage of the internet and electronic devices, with a penetration rate of 90.5% (11). Among medical students at a Mexican public university, 97.6% reported owning a smartphone and 71.5% reported owning a personal computer in a 2019 cohort (12). Notably, second-year medical students at UNAM are in a pre-clinical phase with intensive theoretical coursework (e.g., Physiology) and standardized exams. This context presents high cognitive demands, making it an ideal group to examine whether screen time habits interfere with learning and performance.

Consequently, it is necessary to elucidate the correlation between screen time and cognitive abilities among medical students. Nevertheless, most prior research on digital media and cognition has focused on general cognitive outcomes, without dissecting specific intelligence components (Gc and Gf) or their role in academic performance. These relationships remain largely unexamined in medical student populations, creating a clear gap that this study aims to address. In this study, “digital media exposure” refers to the amount of screen time spent on various activities (quantitative use) rather than the qualitative nature of those activities. We acknowledge that screen time can be utilized in different ways—focused or distracted, educational or recreational—which may differentially impact cognition. Our focus is on the quantitative aspect of usage as measured by time. Within this context, a novel approach employing network analysis offers innovative methodologies for identifying patterns associated with digital media exposure, intelligence, attention, and academic performance. This study aimed to ascertain whether increased exposure to electronic media adversely affects cognitive abilities or academic outcomes. The network approach allowed us to examine local short–distance and systemic long–distance associations among screen time, intelligence, attention, academic performance, and social determinants.

## Methods

### Participants

This study was conducted at the Faculty of Medicine, National Autonomous University of Mexico (UNAM) in Mexico City, which is the largest medical school in the country. The sample consisted of second-year medical students enrolled in the 2010 program, (pre-clinical stage) of the medical curriculum, which emphasizes foundational sciences like Physiology. Participants were selected using a non-probabilistic convenience sampling method during scheduled class times, with no incentives for participation to ensure consistency, data collection took place in 2023. We invited all second-year medical students in the program to participate via announcements and information sessions. A total of 305 students consented and completed the assessments.

### Research design

This study employed an observational, cross-sectional, correlational design. Data were collected at a single point in time to analyze the relationships between academic trajectory, intelligence, electronic media use, and attention. No experimental manipulation was conducted and the study focused on identifying associations rather than causation.

### Measurement of variables

**Figure 1:**
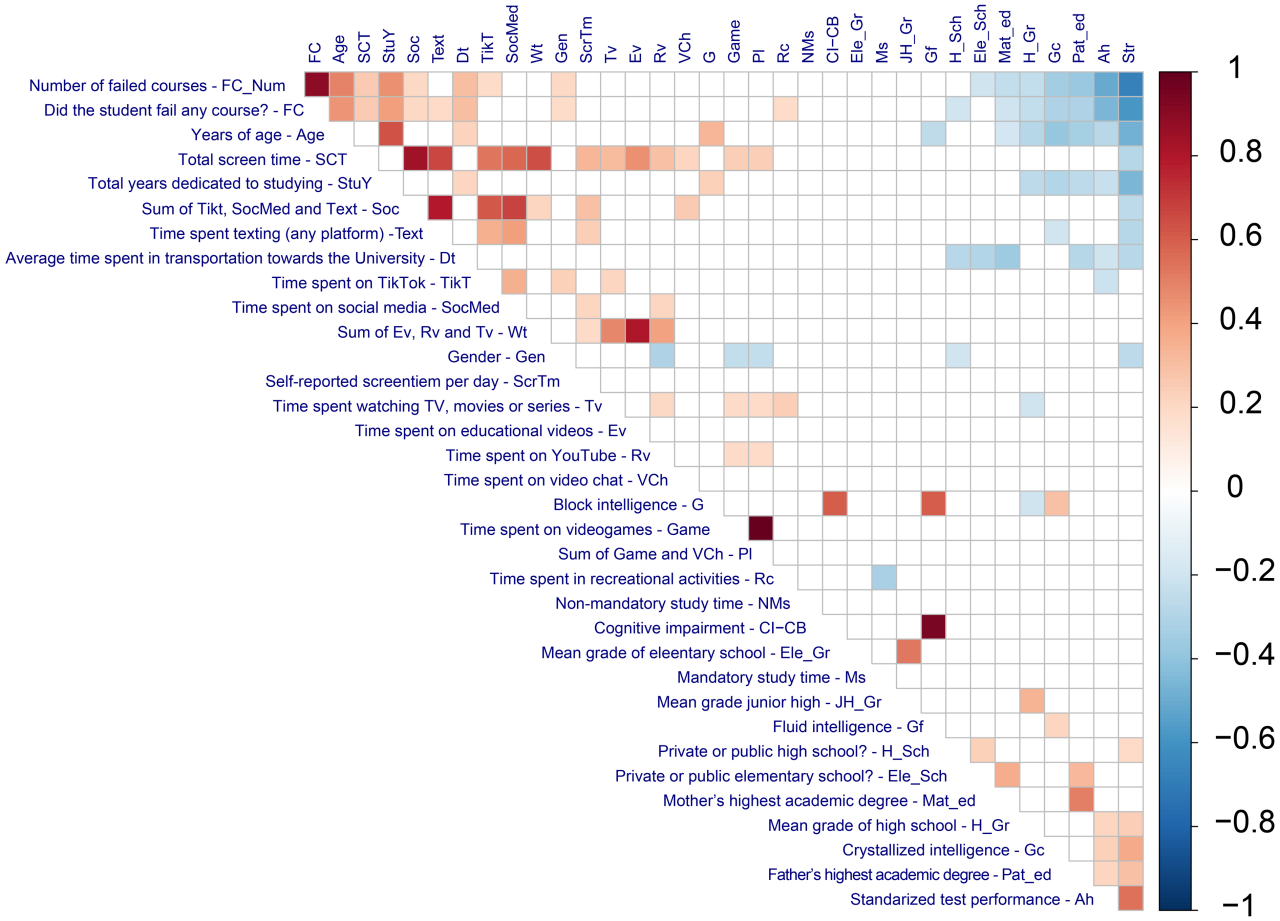
Heatmap of pairwise Spearman correlations among 34 variables. To select statistically significant correlations, a threshold of *p* < 0.001 was applied. Cell color indicates correlation strength and direction (red = positive, blue = negative; deeper color = stronger relationship). SCT = total screen time; Text = texting time; Soc = social media time; Tv = television/video content time; Gf = fluid intelligence; Gc = crystallized intelligence; FC_Num = number of failed courses; Str = standardized physiology test score; Ele_Gr, Jr_Gr, and H_Gr = elementary, junior high, and high school grade averages, etc.

The “socio-academic” category comprised variables related to students’ academic background and status (e.g., age, years of study, number of failed courses), while “early grades” included prior academic performance indicators (such as elementary, junior high, and high school grade averages). The “study-recreation” category captured how students allocate time outside of mandatory academics (e.g., time spent in non-mandatory study or leisure activities). These variables were studied and analyzed were categorized into the following dimensions (see Figure 1):

- Digital behavior
- Videogames
- Study-recreation
- Socio academic
- Early grades
- Intelligence

All participants completed an online sociodemographic survey and provided information regarding their academic trajectory (an official document from the UNAM’s School Administration System) up to the time of the study. Intelligence was assessed using the standardized intelligence test Shipley-2 ^®^ (13). Electronic media use was measured using the Youth Screen Time Survey (14).

These particular variables were selected based on the aims of the study and prior research. The Youth Screen Time Survey targets the most common digital activities among students—watching video content, social communication (messaging/social media), and gaming—providing a broad measure of *digital media use*. We chose the Physiology course’s standardized test scores as the indicator of academic performance because this core course demands integration of knowledge and cognitive skills, making it a representative benchmark for academic achievement in the second year. We narrowed the scope to these constructs (digital use, intelligence, attention, and performance) to maintain a manageable survey length and because they align with our conceptual framework, whereas other potential factors (e.g., podcasts, personality traits) were beyond the scope of this study.

The instruments were administered to groups of 15 students each, ensuring adequate control of the process and direct supervision by the evaluators. Special care was taken to prepare the classroom environment, ensuring that it was free from visual and auditory distractions. These sessions were conducted in a controlled classroom setting by the research team, who provided standardized instructions. All participants gave informed consent, and it was emphasized that participation was voluntary and unrelated to course grades or academic standing (to avoid any coercion). An evaluator was present throughout to answer questions and ensure students were comfortable and not disturbed, adhering to ethical guidelines for testing.

### Academic trajectory

Academic trajectory was assessed using standardized test scores for the physiological course. The physiology course grade was selected because of its evaluation of cognitive dimensions, such as integration and conceptual analysis. The department provides results from three standardized tests conducted every 4 months, with Cronbach’s alpha values of 0.857, 0.898, and 0.907. The average of these three test scores was calculated and used as a variable for standardized test performance (Str).

### Brief intelligence scale test

Cognitive intelligence was evaluated using the Shipley-2 ^®^ test, a psychometric instrument designed to measure various aspects of cognitive intelligence including Gf, Gc, general intelligence, and potential indicators of cognitive impairment. The Shipley-2 ^®^ test comprises two main sections: (I) Vocabulary scale (Gc assessment), in which participants selected synonyms. This section evaluates acquired knowledge such as vocabulary and verbal comprehension, reflecting prior experiences and learning. Gc is closely associated with skills accumulated over time. (II) Abstraction scale (Gf assessment), in which participants completed pattern-related problems. This section measures logical reasoning and problem-solving abilities, which are essential aspects of fluid intelligence that are independent of prior learning and are related to abstract reasoning. The test was administered and analyzed according to the instructions published in the original version, without modifications.

### Screen time survey

The screen time was assessed using the method developed by Sauce et al. (14). The participants were asked to rate their daily use of various applications on weekdays and weekends, including activities such as watching programs and videos, playing video games, sending messages, using social media, and video chatting. The rating scale ranged from “none” to “>4 h,” and means were calculated for both temporal contexts. Based on previous research, screen time was categorized into three main groups: (i) watching content (programs and videos); (ii) socializing (messages, social media, and video calls); and (iii) playing video games.

### Data analysis

The databases were constructed using Microsoft Excel (Microsoft Corporation, Redmond, Washington, United States), and analyses were performed using IBM SPSS Statistics for Windows, Version 25.0 (2017; IBM Corp., Armonk, New York, United States), GraphPad Prism version 9 (Dotmatics, Boston, Massachusetts, United States), and Excel. The Kolmogorov–Smirnov test was used to evaluate normality. Descriptive statistics for the study population included measures of central tendency, dispersion, and frequency. Scatter plots and box-and-whisker plots were generated for data visualization. The hypothesis testing included Pearson’s correlation and linear regression. Additionally, network analysis was conducted in RStudio, Version 2025.05.0 + 496 (Posit Software, PBC), using the igraph package to identify clusters and analyze connectivity patterns among variables, thereby elucidating the underlying complexity of the data. Conditional Uniform Graph Tests (CUG Tests) were conducted to determine the statistical significance of the selected Graph Level Indexes (GLIs) when compared to randomly generated graphs with the same number of edges. CUG tests were performed with 100 Monte Carlo replications and *p*-values were reported. Using the resulting network topology, we generated a mediation model and verified the influence and interactions of the screen time and intelligence variables. The mediation model was implemented in Jamovi 2.7.6 (Sydney, Australia).

### Network construction and analysis

The physiological network construction was based on a previous study (15–17). First, all data were screened for extreme values and normality using the Shapiro–Wilk test. Missing values were imputed using a non-parametric approach with random forests implemented in R, using the missRanger package. No variables had more than 10% of missing values. Data sets were not normally distributed, and ordinal variables were present; consequently, Spearman’s rank correlation was selected as a measure for correlation. To select statistically significant correlations, a threshold of *p* < 0.001 was chosen to account for multiple comparisons and reduce the likelihood of false-positive edges in the network. The Spearman rank correlation coefficient “*ρ*” (rho) was squared to obtain positive values (15).

**Figure 2:**
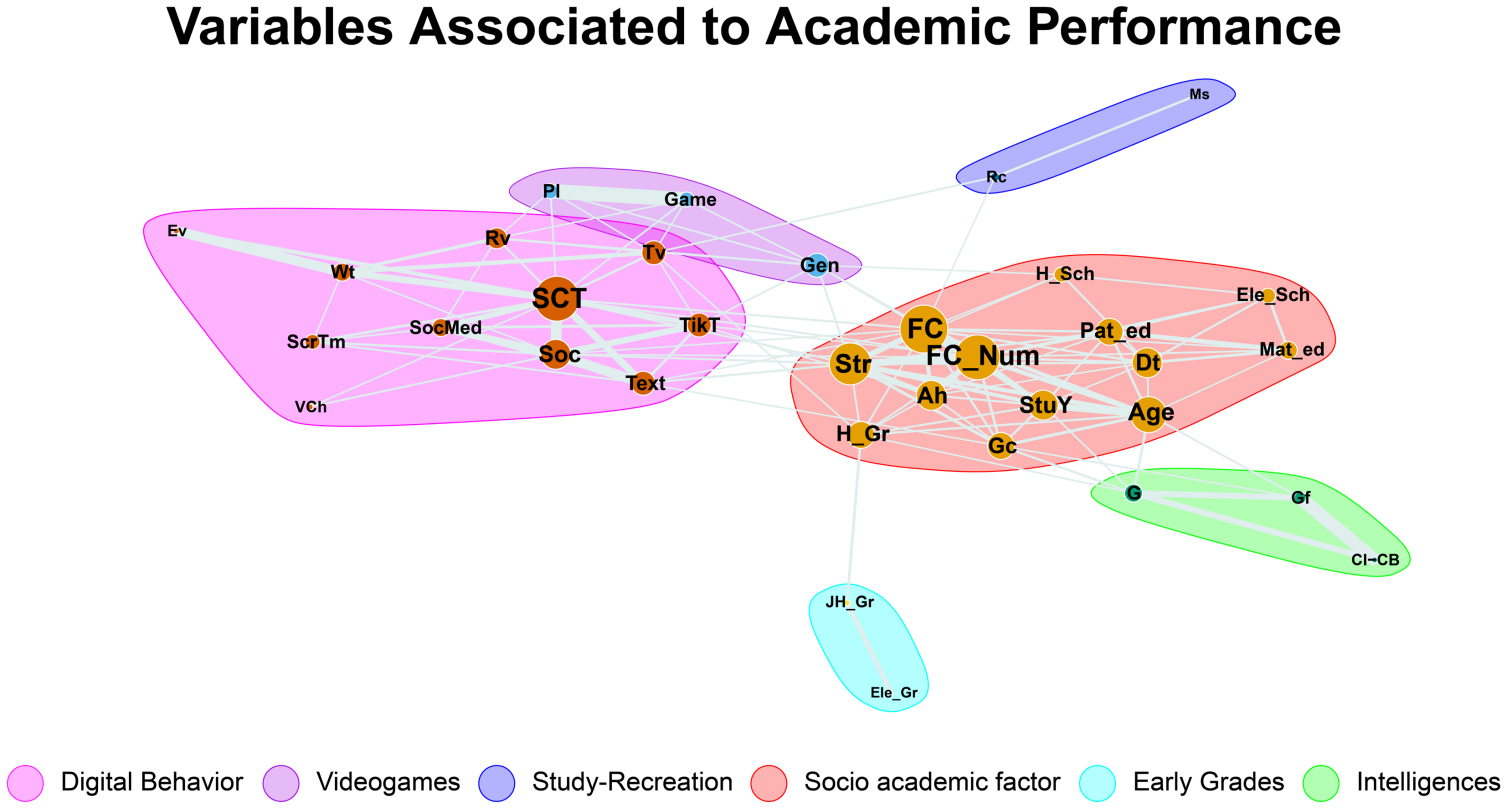
Network of associations between digital behavior, cognitive, and academic variables (34-node network). Each node represents a variable (colored by category: e.g., red for digital use behaviors, blue for intelligence measures, green for academic outcomes, orange for socio-academic factors). Edges connect variables that have a significant correlation (Spearman *ρ*, *p* < 0.001). The network is visualized with a force-directed layout, and six clusters are shaded/highlighted, corresponding to groups of tightly connected variables identified via the Louvain community algorithm. Node size reflects centrality (larger nodes have higher degree/strength, indicating more connections). Notably, the most central nodes were FC_Num (failed courses), SCT (total screen time), Age, and Str (physiology score), indicating these variables bridge multiple connections in the network (see Figure 1 for correlation values; SCT, total screen time; Text, texting time; Soc, social media time; Tv, television/video content time; Gf, fluid intelligence; Gc, crystallized intelligence; FC_Num, number of failed courses; Str, standardized physiology test score; Ele_Gr/Jr_Gr/H_Gr, elementary/junior high/high school grade average, etc.).

An adjacency matrix was constructed using the determination coefficient between each pair of variables (Figure 2), resulting in a weighted and undirected network (Figure 1). Notably, although the squared Spearman’s *ρ* (coefficient of determination) was employed to derive the adjacency matrix weights—thereby ensuring that all edge magnitudes remained positive—the original sign of each correlation was preserved for interpretative purposes. Thus, the network visualizations (the heatmap in Figure 2 and the graph in Figure 1) denote whether each association was positive or negative, even though the corresponding edge weight is derived from ρ^2^. The matrices were rearranged in first principal component order (Figure 2). The resulting graph was plotted using a graphopt layout implemented in the igraph package (Figure 1).

We evaluated the graph topology using network density, transitivity, and average path length. Node centrality was evaluated using the sna package (18) to calculate degree, strength, eigencentrality, and betweenness. Eigencentrality corresponds to the value of each eigenvector inside the graph adjacency matrix and is interpreted as a measure of influence within the network. Betweenness was selected as a measure of intermediation within the network. Eigencentrality and betweenness show different roles that a node may play within the network (19).

To analyze the microstructure of our graph, we ranked each node according to its degree and strength. Nodes within a network can also be ranked according to several centrality definitions (19). Radial and medial centralities were assesses though eigencentrality and betweennes respectively, both values were obtained using the sna package (18).

The vicinity of the correlated variables was delimited via cluster analysis using the Louvain algorithm. Briefly, node groups were determined using the modularity optimization algorithm as calculated in the igraph package. The resulting clusters were delimited using shadows of different colors (Figure 1).

## Results

A total of 305 students participated, including 220 women (66%), 84 men, and one non-binary. The average age of the participants was 20 years (SD = 2 years).

We conducted multiple analyses among all variables separated by gender, and only digital gaming time (Game) was statistically different according to multiple regression analysis.

### Network topology

Regarding our main objective, examining whether prolonged exposure to electronic media negatively influences cognitive abilities, academic trajectories, and social determinants in medical students, we examined only the main connected component of our network. The time spent in Non-Mandatory Activities was not significantly correlated with any other variable in our study, and this single node was removed from the network (data not shown). This indicates that all other variables are systemically related to each other.

Overall, the density of the network was 0.21 (*p* < 0.0001), indicating that only 21% of all possible links were in this network. The network transitivity was 0.5208 (*p* < 0.0001), indicating that 52% of all possible connected triangles among any three individual nodes in the network were present. Average path length was 2.29, which means it takes on average 2.29 “steps” to connect any two nodes from the network. This short path length reflects the relatively small size of the network and an efficient pattern of connections. Despite this, clustering is also apparent in the network. Overall, our network has subgroups of nodes that are substantially connected among themselves, yet this high degree of connection is not maintained throughout the network, hinting at a small-world network akin to biological systems.

Our network comprised 34 variables (nodes) connected by 122 statistically significant correlations (edges) (Figure 1). Our network comprised six main clusters (Figure 1). We observed three distinct groups of variables in our heatmap (Figure 2), we can observe 3 distinct groups of variables. These relate to (1) time spent on digital media, videogames, and social media use (upper left corner); (2) different intelligences and their relationship to age, number of failed exams, and years of study (upper right corner); and (3) the relationship between different intelligences and academic performance (bottom right corner).

When computing the degree of each node, the number of connections for each variable, number of failed exams (FC, FC_Num), time spent using electronics (SCT), grades (Str), and age (Age) were the most connected inside the network. The connectedness of these same nodes was confirmed using a basic centrality measure, eigencentrality. Variables with the highest centrality (i.e., the most interconnected and influential nodes in the network) were the number of failed courses (FC_Num), total screen time (SCT), and age. This shows that these nodes are key variables in our model and have the greatest influence inside the network (these nodes had the greatest number and strength of connections to others), suggesting that they are central to academic success.

On the other hand, the highest betweenness scores were allocated to: Average grade during highschool (H_Gr), number of failed exams (FC_Num), TV time (Tv), texting time (Text), and total social media time (Soc). It is noteworthy that FC_Num ranks among the highest in both eigencentrality and betweenness, indicating that failed courses have a profound impact on academic success, both as a factor by itself and as a mediating factor between different types of intelligence and digital media use. TV time seems to bridge videogame use and overall digital media consumption, while Text and Soc are directly connected to average grades (Str).

The shortest path connecting the intelligence and electronic media clusters passes through texting. Texting had the fifth-highest betweenness among the nodes (51, *p* < 0.38). This indicates that any path connecting the digital behavior cluster and socio-academic factors most likely crossed the text node. Moreover, only texting was significantly associated with a type of intelligence (Gc) but was negatively correlated (*ρ* = −0.20, *p* < 0.01). Therefore, texting is one of the most common digital behaviors and is intricately related to socio-academic factors; yet it seems to hinder or discourage the use of consolidated intelligence and memory.

Our network highlights the time-dependent relationship between grades throughout academic development. Elementary school grades (Ele_Gr) are related to junior high grades (Jr_Gr), but not to high school (H_Gr) or university grades (Ah, Str). Moreover, junior high grades are related to high school grades but not to others. This suggests that previous term grades can be a predictor of academic performance, but only in a short timeframe. This highlights the importance of good grades, even early in school.

Electronic media, regardless of the form, was highly connected among themselves, but only total screen time, texting, and Tik Tok time seemed to have a relationship with the number of failed courses (FC, FC_Num).

Academic performance in university (Str, Ah) shows a relationship with Tik Tok use and Texting, yet further research is needed to clarify its relationship and if it is directly responsible for hindering academic development effect.

### Clusters

Louvain’s algorithm exhibited a high modularity, with a value of 0.56. Six clusters were identified in the network (Figure 1). The first cluster (red) included grades, crystallized intelligence (Gc), transportation, age, and parents’ degree of study. This cluster has most of the nodes with high eigencentrality in the network, including grades (Ah), number of failed exams (FC, FC_Num) and average grade (Str). A key node with both high eigencentrality and highest betweenness is the number of failed exams (FC_Num).

The second cluster (pink) includes the time spent on all forms of electronic media (texting, social media, videos, and television). No nodes in this cluster have high eigencentrality, yet betweenness is high for time spent on TV, texting, and Tik Tok.

The third cluster (purple) includes the gender and time spent on videogames. It is apparent that there is a broad relationship between gender and playing video games. None of these clusters had nodes with high centrality or betweenness.

The fourth cluster (navy blue) relates mandatory study time (Ms) with time spent in recreational activities. Interestingly, Mandatory study time was not related to time spent on electronic media or academic success. None of these clusters had nodes with high centrality or betweenness.

The fifth cluster (green) included cognitive impairment (CI-CB), fluid intelligence (Gf), and intelligence (G), excluding crystallized intelligence (Gc). It is noteworthy that intelligence (G) was closely related to age, years dedicated to study, and high school grades, but not to other metrics of academic achievement or electronic media use.

Finally, the sixth cluster (sky blue) solely relates elementary school grades to junior high grades. It seems apparent that there is a sequential relationship for academic performance, spanning from elementary school to university. None of these clusters had nodes with high centrality or betweenness.

**Figure 3:**
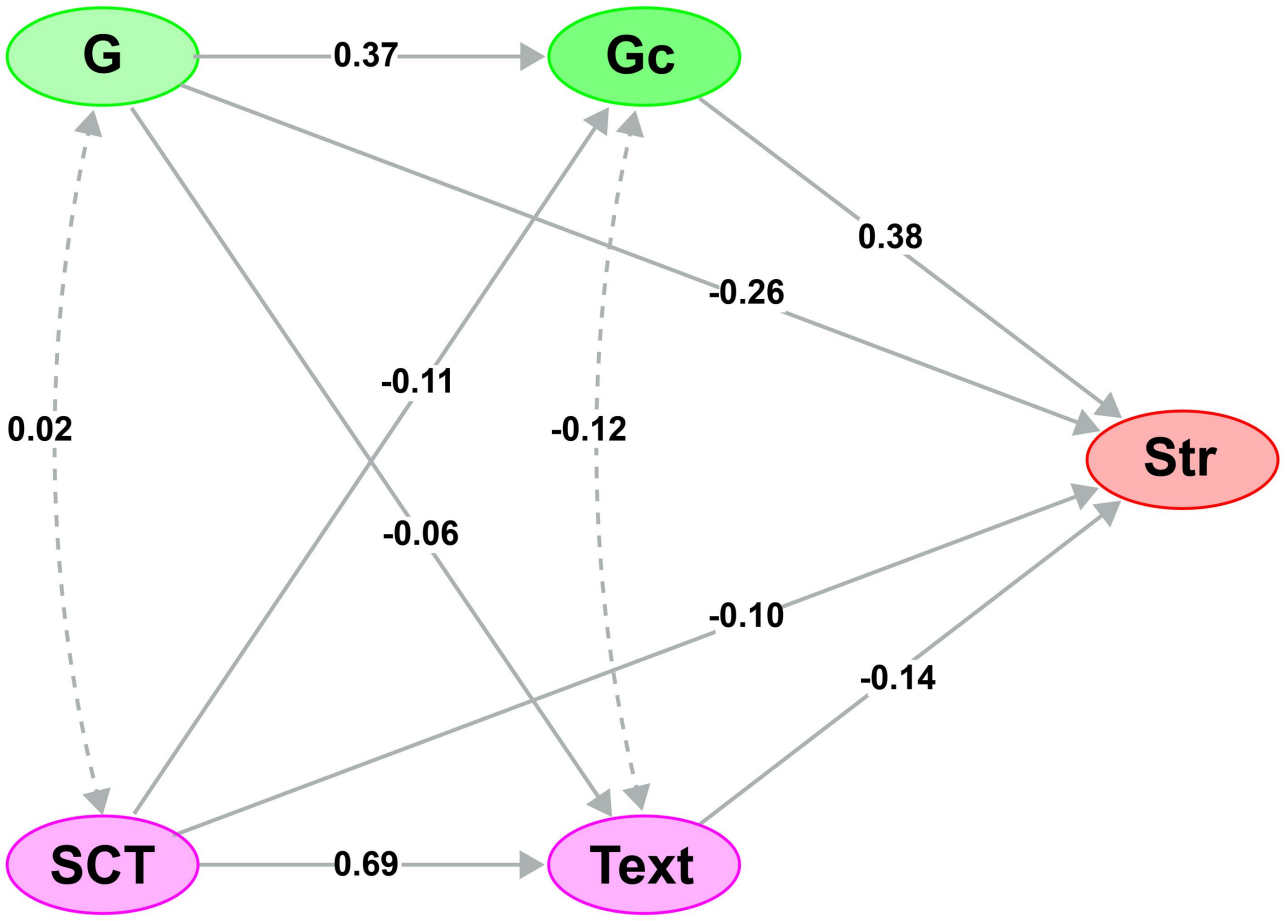
Mediation model linking intelligence, screen time, and academic performance. This structural model examines how screen time and specific digital behaviors may indirectly affect academic performance (Str) through intelligence factors. Solid arrows indicate significant standardized regression paths (with *β* coefficients shown next to each arrow). For example, higher crystallized intelligence (Gc) is associated with better performance, whereas greater total screen time (SCT) and more texting (Text) are associated with poorer performance (all *p* < 0.05). Fluid intelligence (Gf) is included as a mediator that could be influenced by screen time and in turn affect performance. Dotted arrows represent covariance between Gc and Gf (indicating these two intelligence measures are correlated, as expected, but this is not a causal path). This model is cross-sectional, so while it suggests possible indirect effects (e.g., excessive screen time → lower Gc → lower performance), temporal ordering is not confirmed (see Figures 1, 2).

To deepen our understanding of the effect of intelligence and Total Screen Time on Academic performance, we conducted a mediation analysis with only five variables: Intelligence (G), Crystallized Intelligence (Gc), Total Screen Time (SCT), Texting (Text), and Standardized Physiology test results (Str). In Figure 3, solid arrows indicate direct pathways (regression coefficients) between variables, whereas dotted arrows represent covariance relationships between the mediator variables. All Beta coefficients (*β*) are presented in Table 1. We observed that higher crystallized intelligence (Gc) was associated with better academic performance (*β* = 0.38), whereas greater total screen time (SCT) and more texting were associated with lower performance (*β* = −0.10 and *β* = −0.14, respectively).

**Table 1:** Indirect and total effects.

Type	Effect	Estimate	SE	95% C. I. (a)		*β*	*z*	*p*
				Lower	Upper			
Indirect	SCT ⇒ Text ⇒ Str	**−1.474**	**0.7509**	**−2.9458**	**−0.00246**	**−0.09522**	**−1.96**	**0.05**
	SCT ⇒ Gc ⇒ Str	−0.634	0.3253	−1.2714	0.00378	−0.04094	−1.95	0.051
	G ⇒ Text ⇒ Str	0.14	0.1141	−0.0839	0.36326	0.00902	1.22	0.221
	G ⇒ Gc ⇒ Str	**2.157**	**0.4421**	**1.2906**	**3.02365**	**0.13933**	**4.88**	**<0.001**
Component	SCT ⇒ Text	**1.076**	**0.0654**	**0.9479**	**1.20427**	**0.68516**	**16.45**	**<0.001**
	Text ⇒ Str	**−1.37**	**0.6928**	**−2.7277**	**−0.01205**	**−0.13897**	**−1.98**	**0.048**
	SCT ⇒ Gc	**−1.662**	**0.8182**	**−3.2655**	**−0.0584**	**−0.10766**	**−2.03**	**0.042**
	Gc ⇒ Str	**0.381**	**0.0554**	**0.2728**	**0.48992**	**0.38029**	**6.89**	**<0.001**
	G ⇒ Text	−0.102	0.0654	−0.2301	0.02624	−0.06491	−1.56	0.119
	G ⇒ Gc	**5.656**	**0.8182**	**4.0526**	**7.25969**	**0.36639**	**6.91**	**<0.001**
Direct	SCT ⇒ Str	−1.607	1.0796	−3.7232	0.50858	−0.10382	−1.49	0.137
	G ⇒ Str	**−4.092**	**0.8457**	**−5.7499**	**−2.43463**	**−0.26433**	**−4.84**	**<0.001**
Total	SCT ⇒ Str	**−3.715**	**0.8554**	**−5.3919**	**−2.03861**	**−0.23998**	**−4.34**	**<0.001**
	G ⇒ Str	**−1.795**	**0.8554**	**−3.4721**	**−0.11885**	**−0.11597**	**−2.1**	**0.036**

Confidence intervals computed with method: Standard (Delta method). Betas are completely standardized effect sizes. Bold values indicate statistically significant effects (*p* < 0.05). Confidence intervals were computed using the standard (delta) method. *β* values represent completely standardized effect sizes.

## Discussion

The results showed a significant inverse relationship between screen time and academic performance, with a moderate negative association (*r* ≈ −0.24, *p* < 0.01), which is consistent with the findings in other educational populations (20, 21). In a longitudinal study of Canadian schoolchildren, each additional hour of daily media use was associated with decreased performance on standardized reading and math tests (22). In fact, recent literature synthesized by meta-analysis confirms a small but consistent adverse effect of excessive smartphone use on academic performance (*r* ∼ −0.10, *p* < 0.01), particularly pronounced in younger students (23, 24). Among university students, specifically in medicine, it has been consistently reported that students with signs of smartphone addiction (25) have significantly lower grades along with impaired study and health habits (8, 26). Taken together, these data reinforce the association observed in our study, where high use of electronic devices, estimated at ~7 h per day, is related to lower measurable academic performance on standardized tests.

This link suggests that digital overexposure may be associated with reductions in critical cognitive components for learning. Our findings showed that, although crystallized intelligence (accumulated knowledge, *Gc*) remained stable regardless of screen time, fluid intelligence (*Gf*) and general cognition were reduced with increased media use. This suggests that excessive digital stimuli would not immediately erode acquired knowledge but would compromise the ability to effectively apply that knowledge in new problem-solving environments (27, 28). Even students with a large body of knowledge may not translate this into performance if their attentional and executive resources are overloaded (27, 29). For example, it has been documented that greater nighttime exposure to screens in young adults is associated with lower cognitive domain scores such as working memory, sustained attention, and processing speed (30, 31). In terms of information processing models, attention acts as an input filter to the cognitive system. If attentional filtering is compromised by constant distractions (notifications, rapid changes in content), the extrinsic load on working memory increases, and the portion of mental capacity available for deep processing of academic material decreases (32, 33). Our data precisely support this dynamic: the excessive consumption of digital media could “divert” part of the cognitive resources toward irrelevant stimuli, leaving less availability for the coding and consolidation of academic information, with a consequent negative impact on exam performance.

A novel finding of this research is the identification, through network analysis, of certain digital behaviors as key link nodes between screen use and academic results. In particular, the time spent on instant messaging (e.g., texting) and short-form video platforms (such as TikTok) have emerged as bridges connecting the sphere of recreational electronic use with the sphere of academic achievement. This indicates that such activities could act as intermediate factors that intensify the detrimental effect of excessive screen use on performance, possibly through the fragmentation of the student’s cognitive structure. In other words, applications of ephemeral social interaction and short videos, characterized by highly dynamic content and immediate gratification, seem to introduce constant interruptions in attentional flows, preventing the formation of an integrated network between students’ knowledge, concentration, and practical application (34). This fragmentation of the “cognitive network” manifests itself; for example, in the fact that a student’s high *Gc* fails to counteract distractions, a student may possess a large vocabulary or solid conceptual domains, but if he continuously alternates between studying and reviewing messages or video feeds, his performance almost inevitably declines (8, 35). Literature supports this interference mechanism. Studies using electrophysiological recordings have shown that addiction to short videos on mobile phones is associated with decreased prefrontal executive control and attentional self-regulation (36). Similarly, multitasking involving social media has been shown to generate significant cognitive distractions. In college students, the practice of simultaneously alternating academic tasks and social applications raises the level of mental distraction and mediates a drop in grades (37). Analyses of psychological networks in adolescents suggest that problematic screen use plays a central role in the interactions among cognitive, emotional, and academic factors, presenting high centrality and influence within the system. For example, Xu et al. identified that device addictive behavior acts as a connecting nucleus that exacerbates mental difficulties and affects general cognitive function in youth development networks (38, 39). These comparable results support our identification of messaging and short videos as critical points of disruption, which are seemingly innocuous or trivial activities that, however, facilitate the constant insertion of distracting elements within the student’s cognitive circuit, “breaking” the continuity necessary for deep learning and optimal performance.

From a critical perspective, our findings highlight that not all screen use affects the population studied in the same way, the quality and type of digital interaction matter. The fact that certain facets of intelligence (e.g., crystallized intelligence) do not decline suggests that moderate use focused on academic or informational content may not be detrimental and may even provide resources (e.g., quick access to information). It has been proposed that educational and interactive digital content may offer specific cognitive benefits to young populations (22). However, our data and external evidence indicate that the problem arises with the excessive amount and fragmenting nature of popular digital content. Prolonged and mainly recreational consumption—social networks, short videos, and continuous messaging—tends to displace activities conducive to performance (focused study and adequate rest) and to induce habits of superficial attention. This imbalance translates into mental fatigue, less memory consolidation, and ultimately, lower grades (40). It should be noted that the magnitude of the observed effect, although significant, was moderate, consistent with the notion that academic performance is a multifactorial phenomenon. In addition to the influence of screens, variables such as intrinsic motivation, study strategies, teaching quality, psychosocial support, chronotype, and sleep habits are involved. It is possible that certain highly capable students may be able to partially mitigate the negative effects of digital multitasking (e.g., by applying self-regulation techniques), while more cognitively vulnerable students may suffer a greater impact. These individual differences may partly explain why some previous studies found no significant effects of smartphone use on academic grades (41). However, the convergence of recent empirical evidence tips the balance toward recognizing that, on average, excessive use of devices constitutes a risk factor for suboptimal performance. Therefore, it is prudent to address this phenomenon from public health and educational perspectives.

It is worth noting that our use of network analysis provided a novel perspective beyond what traditional regressions offer. This approach allowed us to identify complex interdependencies and key “hub” variables (like texting and short-video use) that might have been overlooked in a standard linear analysis. By mapping these relationships, we uncovered intermediate nodes that could be targets for intervention to improve academic outcomes.

### Limitations

First, the cross-sectional and observational design of the study precludes establishing any causal relationships or temporal order between screen time, cognitive function, and academic performance. This means that even though we tested a mediation model, we cannot confirm temporal precedence of the mediators; the associations should be interpreted as correlational. Second, the sample was limited to second-year medical students from a single institution (UNAM), which may affect the generalizability of the findings to other populations or settings. Importantly, our participants were in a pre-clinical year; their study habits and cognitive demands differ from those of clinical-year students or other disciplines, so digital media impacts might not be identical in those groups. Third, academic performance in this study was represented by a single course’s standardized exam score (Physiology). Fourth, the study relied on self-reported screen time, and accurately quantifying digital media use is challenging. Self-report measures may introduce recall bias or underestimation/overestimation of actual use. Fifth, in our network analysis we included some composite variables (e.g., total screen time, overall intelligence score) together with their constituent variables (specific app use measures, Gf and Gc). This part–whole inclusion can inflate certain network metrics, such as eigencentrality, because an aggregate node will naturally correlate with its components. We opted to retain these composites for completeness, but this redundancy should be kept in mind when interpreting the network structure.

This is a narrow measure of achievement; students’ abilities can vary across different subjects, and a single-course grade may not reflect their overall academic performance. Using a composite metric or multiple course outcomes in future studies would provide a more comprehensive view. Future research studies should employ longitudinal designs to track how digital media use influences cognitive and academic outcomes over time. Following students across multiple years and incorporating performance in multiple courses would help establish temporal sequences and determine long-term effects. Expanding the sample to other cohorts or institutions (including clinical-year students and different educational settings) will improve generalizability. Additionally, using objective measures of digital activity (such as device usage logs) alongside self-reports can provide more accurate assessments of screen time. Applying similar network-analytic approaches in these studies could further illuminate complex mediating pathways between digital habits and academic success, and ultimately inform interventions. This line of research can guide the development of strategies to help students balance digital engagement with learning—for instance, educational programs or tools to mitigate the distracting aspects of technology while leveraging its benefits for study.

## Data Availability

The datasets presented in this study are publicly available in online repositories. The original dataset can be accessed at Zenodo: https://doi.org/10.5281/zenodo.17486080. Further inquiries can be directed to the corresponding author.
